# A minimum estimate for the incidence of gastric cancer in Eastern Kenya

**DOI:** 10.1054/bjoc.2001.1994

**Published:** 2001-09-01

**Authors:** G McFarlane, D Forman, F Sitas, G Lachlan

**Affiliations:** Department of Surgery, Crosshouse Hospital, Kilmarnock, KA2 0BE, UK; Centre for Cancer Research, University of Leeds, Cookridge Hospital, Arthington House, Leeds, LS16 6QB, UK; National Cancer Registry, Cancer Epidemiology Research Group, P.O. Box 1038, Johannesburg, 2000, South Africa; Belford Hospital, Fort William, PH33 6BS, UK

**Keywords:** gastric cancer, incidence, *Helicobacter pylori*

## Abstract

We documented available information concerning incident cases of gastric cancer in part of Kenya's Eastern Province between 1991 and 1993. By reviewing the records of all major health facilities in the area, 200 cases of gastric carcinoma were found giving an annual average crude incidence rate of 7.01 per 100 000 males and 3.7 for females (world age-standardised rates, 14.3 for males and 7.1 for females). There is likely to be underascertainment of cases especially among those aged over 65 years. Previous incidence estimates for the same area of Kenya were reviewed and a 10-fold increase in the recorded indirectly standardised incidence rate between the periods 1965–70 and 1991–93 was noted but this may be due to improved diagnostic facilities. The recent rates in this part of Kenya are comparable to Eastern European rates and similar to those recorded in other highland regions of Africa. © 2001 Cancer Research Campaign


*British Journal of Cancer* (2001) **85**(9), 1322–1325
© 2001 Cancer Research Campaign
doi: 10.1054/ bjoc.2001.1994, available online at http://www.idealibrary.com on IDEAL

http://www.bjcancer.com

### A minimum estimate for the incidence of gastric cancer in Eastern Kenya

**G McFarlane[1], D Forman[2], F Sitas[3] and G Lachlan**

[1]Department of Surgery, Crosshouse Hospital, Kilmarnock KA2 0BE, UK; [2]Centre for Cancer Research, University of Leeds, Arthington House, Cookridge Hospital, Leeds LS16 6QB, UK; [3]Head, National Cancer Registry, Director, MRC/SAIMR/CANSA/WITS, Cancer Epidemiology Research Group, P.O. Box 1038, Johannesburg 2000, South Africa; [4]Belford Hospital, Fort William, PH33 6BS, UK

**Summary** We documented available information concerning incident cases of gastric cancer in part of Kenya's Eastern Province between 1991 and 1993. By reviewing the records of all major health facilities in the area, 200 cases of gastric carcinoma were found giving an annual average crude incidence rate of 7.01 per 100 000 males and 3.7 for females (world age-standardised rates, 14.3 for males and 7.1 for females). There is likely to be underascertainment of cases especially among those aged over 65 years. Previous incidence estimates for the same area of Kenya were reviewed and a 10-fold increase in the recorded indirectly standardised incidence rate between the periods 1965–70 and 1991–93 was noted but this may be due to improved diagnostic facilities. The recent rates in this part of Kenya are comparable to Eastern European rates and similar to those recorded in other highland regions of Africa. © 2001 Cancer Research Campaign

**Keywords**: gastric cancer; incidence; *Helicobacter pylori*

The role of *Helicobacter pylori* infection in the development of gastric cancer has been questioned partly because of the high levels of infection in much of Africa and apparently low rates of gastric cancer (Holcombe, 1992; Segal et al, 1998). There are, however, few established population-based cancer registries (Chokunonga et al, 1999), and data obtained from these and other sources have recognised deficiencies.

Following a clinical impression that the number of gastric malignancies diagnosed at the Presbyterian Church of East Africa (PCEA) hospital at Chogoria was relatively high, a retrospective survey was undertaken in May 1994 for the years 1991–93 to provide a minimum estimate of incidence for the Greater Meru region of Eastern Kenya from data collected from all 8 hospitals that served the area. The resulting figures were compared with rates available from surveys in the same region in earlier years.

### METHODS

Greater Meru is in Kenya's Eastern Province and has a total population of about 1.2 million. It is divided into 4 administrative districts, Meru Central, Meru South, Meru East and Meru North. In the 8 hospitals that served the area for the period 1991–93 all available theatre books, endoscopy record books and ward admission books were examined and inpatient notes obtained where possible to clarify the diagnosis. In addition, a card system based on the WHO classification of disease assisted case finding in one District Hospital (Isiolo) and computer records of discharge diagnoses were searched at PCEA Chogoria Hospital, the largest in the area. Death certificates are rarely completed in rural Kenya and were therefore not used to search for cases. The resulting database was searched to delete duplicate records, as some patients had been admitted on more than one occasion during the course of their illness, not necessarily at the same hospital.

At hospitals other than Chogoria, the age of the patient was often recorded as 'Adult'. As it is usually patients over the age of 45 years who have little idea of their age, their age was assigned as follows: Cancer patients over 45 years old who had presented to Chogoria Hospital, and who all had their age recorded, were analysed to ascertain the proportion in each 5 year age group. The patients from other hospitals where the age was unknown were then assigned to an age group over 45 years according to these proportions, for males and females separately.

From scanty notes available at some institutions, the reliability of data might be questioned. In order to test the accuracy of the overall figures for the area, patient addresses were recorded to identify those from the Chogoria Hospital catchment area covering an estimated population of 434 000 in 1992. This smaller area consisted of Meru South and East Districts plus the Abogeta, Nkuene and Igoji Divisions of Meru Central where Chogoria Hospital clinics and an extensive community-based health programme had been established. There has thus been a drive over the years to improve access to health care services in the catchment area of the hospital and the incidence among patients living near Chogoria has been used to give a baseline comparison for the rates observed elsewhere in Greater Meru. The ethnic group composition, and diet of the population did not vary from district to district.

Population baseline data from the 1989 census were obtained from the District Population Officer in Meru Town. The crude death rate for the area in 1979 was 1.3% and the crude birth rate 5.2% giving an annual growth rate of 3.9%. This has now dropped to around 3.16% particularly in the Chogoria Hospital catchment area where there is a high uptake of family planning services. Migration in and out of the area is fairly constant at 1% each way.

*Received 23 January 2001*
*Revised 30 May 2001*
*Accepted 5 June 2001*

*Correspondence to:* G McFarlane

An annual growth rate of 3.16% was therefore used to calculate population figures in Greater Meru for the years 1991 to 1993. Crude incidence rates were determined, world age-standardised rates (WASR) calculated by the direct method (Smith, 1992) and the cumulative rate (0–64) ascertained (Plummer, 1997).

For the period 1975 to 1988, numbers of gastric cancer cases presenting to Chogoria Hospital were available from a previous search of ward books, surgical and medical records, death certificates and gastroscopy records carried out during 1988 (Sitas, 1992). Further information for the period 1965–74 was available from part of a larger survey (P Cook-Mozzafari and D Burkitt, unpublished data). Data from the 1969 and 1979 Kenyan National Census (Ministry of Finance 1970, 1980) were used to estimate the age-specific population sizes for Greater Meru from 1965–82. Results of the 1989 census were available to calculate rates for the area from 1983 to 1993. The only age-adjusted incidence rates available from 1965 to 1982 were calculated by the indirect method (Sitas, 1992) and corresponding indirect rates were calculated for the periods 1982–88 and 1991–93 to examine the trend over the 28-year period. Expected numbers were derived from average age-sex-specific rates from 4 African registries that during the 1950s and 60s had meticulous surveys of cancer incidence in defined populations (Johannesburg and Natal, South Africa; Ibadan, Nigeria; Kayondo, Uganda (Doll et al, 1966, 1970)) The ratio of observed to expected numbers of gastric cancer cases was multiplied by the average crude incidence rate for gastric cancer of 7.09 per 100 000 from the above registries to yield an indirectly adjusted incidence rate for each time period.

### RESULTS

The population of Greater Meru was 1 144 594 at the 1989 census and had risen to over 1.2 million by 1991 with 35% living within the Chogoria Hospital catchment area. 60% of the population was under 20 years of age.

A total of 200 patients presented with a new diagnosis of gastric cancer in Greater Meru in the 3-year period, of which 87 presented to Chogoria Hospital, with the remainder seen at 5 of the other 7 hospitals (Table 1). The age range was from 25 to 87 years with 39 patients simply recorded as ‘Adult’. 130 patients were male, giving a male to female ratio of 1.8:1. The method of diagnosis for each patient is recorded in Table 2, for the whole group and the Chogoria Hospital catchment area. Only 9% of all cases were confirmed by histology. In 25% of patients, most of whom were at hospitals other than Chogoria, the method of diagnosis was not clear from the ward admission book or case sheet.

**Table 1** Gastric cancer cases residing in Greater Meru occurring between 1/1/91 and 31/12/93

| District | Hospital | Cases |
|---|---|---|
| Meru South | Chuka District Hospital | 0 |
| | PCEA Chogoria Hospital | 87 |
| Meru Central | Meru District Hospital | 28 |
| | Consolata Hospital, Nkubu | 47 |
| Meru North | Consolata Hospital, Tigania | 16 |
| | Maua Methodist Hospital | 1 |
| Isiolo District | Ishiolo District Hospital | 21 |
| Embu District | Consolata Hospital, Kyeni | 0 |

Using the mid-1992 population figures, the crude incidence rate per 100 000 for gastric cancer was found to be 7.0 (95% Confidence Interval (CI) 5.8–8.2) for males and 3.7 (CI 2.8–4.5) for females over the 3-year period. The rates for the Chogoria Hospital catchment area for males and females combined for the 3 years are given in Table 3 for comparison with the rest of the area.

The world age-standardised rates for males and females were 14.3 (CI 11.8–16.8) per 100 000 for males and 7.1 (CI 5.4–8.8) for females for the years 1991 to 1993. Cumulative rates (0–64) for the same period were 1.07 (standard error (SE) = 0.19) for males and 0.62 (SE = 0.14) for females.

Table 4 shows the indirectly adjusted incidence rates for gastric cancer for the region from 1965 to 1993. It should be noted that the figures from 1965 to 1988 will be an underestimate as case finding was limited to PCEA Chogoria Hospital.

**Table 2** Method of diagnosis of gastric cancer

| Diagnostic method | Chogoria catchment area | Rest of Meru Central & Meru North Districts | All cases |
|---|---|---|---|
| Laparotomy | 36 (58%) | 67 (49%) | 103 (52%) |
| Endoscopy | 23 (37%) | 25 (18%) | 48 (24%) |
| Barium meal | 0 (0%) | 7 (5%) | 7 (3%) |
| Histological confirmation | 8 (13%) | 10 (7%) | 18 (9%) |
| Clinical | 12 (19%) | 6 (4%) | 18 (9%) |
| Unknown | 4 (6%) | 46 (33%) | 50 (25%) |
| Total number of cases | 62 | 138 | 200 |

**Table 3** Comparison of incidence rates per annum, males and females combined (95% confidence intervals) for gastric cancer within Kenya’s Eastern Region

| | Chogoria catchment area | Rest of Meru Central & Meru North Districts | Greater Meru |
|---|---|---|---|
| Number of cases (over 3 years) | 62 | 138 | 200 |
| Population (mid-1992) | 434 033 | 822 534 | 1 256 567 |
| Crude rate (per 100 000) | 4.8 (3.6–5.9) | 5.6 (4.7–6.5) | 5.3 (4.6–6.0) |
| Age-standardised (world) rate (per 100 000) | 9.4 (7.0–11.8) | 11.2 (9.1–13.3) | 10.6 (8.0–13.1) |
| Cumulative rate (0–64) | 0.76 (0.33–1.19) | 0.82 (0.51–1.13) | 0.81 (0.57–1.05) |

**Table 4** Indirectly adjusted incidence rates 1965 to 1992, males and females combined

| Year | Number of cases | Rate (per 100 000) |
|---|---|---|
| 1965–70[a] | 17 | 1.0 |
| 1971–76[a] | 45 | 2.1 |
| 1977–82[b] | 32 | 1.4 |
| 1983–88[b] | 177 | 6.0 |
| 1991–93 | 200 | 11.0 |

[a](P Cook-Mozzafari and D Burkitt, unpublished data; Sitas, 1992)
[b](Sitas, 1992)

© 2001 Cancer Research Campaign

### DISCUSSION

Reliable estimation of cancer incidence rates in rural Africa is extremely difficult to obtain, with few established cancer registries. Registration of deaths is often far from complete, and in some registers, many cancer deaths are recorded as 'unknown primary', indicating that the quality of data is likely to be poor with many poor and elderly patients dying at home without seeking medical services (Smith, 1992). Completeness may be better, however, in some urban African populations (Parkin et al, 2001).

The world age-standardised rates observed in Greater Meru for the years 1991–93 are above those in most of the USA, and are similar to Western Europe (Parkin et al, 1997). The cumulative rate (0–64) has the advantage of being independent of any standard population (Plummer, 1997) and permits comparisons of incidence that omit the elderly who may be underrepresented among those who are diagnosed in hospital. Using this method, the rates of 1.07 for males and 0.62 for females for Greater Meru are above those in US whites (typically 0.3 for males and 0.1 for females), US black populations and Western European nations, similar to Eastern European nations but below rates for Central and South America (averaging approximately 1.6 for males and 0.9 for females) and well below parts of China and Japan (Parkin et al, 1997). Assessed in this way, gastric cancer in this part of Kenya is not uncommon.

The reliability of the data presented here may be questioned due to the low proportion of histological diagnoses. However, registries relying on autopsy reports, with a high proportion of cases confirmed by histology, will inevitably underestimate the true incidence of cancer in Africa. Cancer is often seen by the clinician in this region at an advanced stage, when histological confirmation is of academic interest only, and a needless burden for the patient. Laborious manual procedures had to be adopted in this study to find the necessary information. Even then, the lack of histological confirmation and poor information on such basic variables as age, means that there are considerable uncertainties in the results despite the methods for estimating of missing age information described above. It is also known that in poorer countries many elderly patients die at home without seeking medical services (Smith, 1992) and the age-specific rates in the present study suggest that there is indeed under-diagnosis among those over 65 years of age (assuming most of the clinical diagnoses are genuine stomach cancers).

The consistency of the data presented here is supported by a smaller survey of a population around Chogoria Hospital where access to medical services is better than in many other rural areas of Kenya. Within this group, 75% of cases presenting were diagnosed by laparotomy or endoscopy carried out by a qualified surgeon. Figures from the rest of the survey area were slightly higher than for the Chogoria catchment area suggesting that case finding was at least as complete as around Chogoria hospital. Coverage of hospitals throughout the Meru Districts and beyond, with recording of the addresses of the patients, weighs against high rates based on data from Chogoria Hospital merely reflecting the attraction of patients from more distant districts who had heard of the specialist interest of the hospital.

A summary of the currently available incidence data for gastric cancer in Africa is shown in Table 5. With the obvious exception of Mali, higher rates of cancer tend to be found in populations residing at higher altitude. Studies of the relative frequencies of cancers, for example in Southern Rwanda (Ngendahayo and Parkin, 1986; Newton et al, 1996) at an altitude of 1500–2000 m and the Kilimanjaro area of Tanzania (Lauren and Kitinya, 1986) at an altitude of 1000 m have supported this finding. Relative frequency data from the 1950s and 1960s suggest that the probable high incidence in these areas is long-standing (Cook and Burkitt, 1971) and that it extended also into Burundi and neighbouring Zaire as well as Tanzania. Gastric carcinoma has also been noted to be relatively common in an endoscopic series from the Bale Highlands of Southern Ethiopia (Madebo et al, 1994). It has been suggested that the increased incidence in highland regions may be due to volcanic soils (Kitinya et al, 1988) or to an interaction between *Helicobacter pylori*, the immune response and malaria (Blaser, 1993) but the effect might also be the result of better soils and climate at higher altitude promoting improved socio-economic status, and leading to better access to health care. The unusually high incidence of stomach cancer found in Mali has been attributed to the frequent use of sauces containing smoked or dried fish which may be rich in nitrosamines (Bayo et al, 1990).

Although the figures given in Table 4 suggest a considerable rise in the incidence of gastric cancer in the survey area over the past 27 years, it is difficult to know whether this is real or apparent. A qualified surgeon arrived in Chogoria for the first time in 1982 and a gastroscopy service was established in 1984. During this period, ease of communication and the socio-economic status of the area were also improving. In Zimbabwe, 2 hospital-based

**Table 5** World age-standardised incidence rates of gastric cancer and altitude of various African populations. Standard error in brackets where available

| Location | Year | Altitude (metres) | WASR males | WASR females |
|---|---|---|---|---|
| Mali, Bamako[a] | 1988–92 | 100 | 19.6 (1.7) | 11.1 (1.3) |
| Kenya, Meru | 1991–93 | 1500 | 14.3 (1.3) | 7.1 (0.9) |
| Algeria, Setif[a] | 1990–93 | 1200 | 14.4 (1.4) | 3.5 (0.8) |
| Zimbabwe, Harare[b] | 1993–95 | 1300 | 12.3 | 11.0 |
| Guinea, Conakry[c] | 1992–95 | 50 | 6.1 | 5.7 |
| Uganda, Kyadondo Country[d] | 1991–94 | 1200 | 4.7 (1.0) | 3.2 (0.7) |
| Ivory Cost, Abidjan[e] | 1995–97 | 50 | 3.3 | 4.5 |
| The Gambia[e] | 1988–97 | 50 | 2.3 | 1.9 |

[a](Parkin et al, 1997). [b](Chokunonga et al, 2000). [c](Koulibaly et al, 1997). [d](Wabinga et al, 2000). [e](Echimane et al, 2000).

 *© 2001 Cancer Research Campaign*

studies undertaken 20 years apart point towards a real rise in the incidence of gastric cancer (Dent, 1989). However, in a recent population-based study in Uganda, a substantial rise in the incidence of gastric cancer was attributed to improved access to medical services and increased availability of gastroscopy (Wabinga et al, 2000), while in Soweto, South Africa, no increases in the relative frequency of gastric cancer have been detected since 1948 (Segal et al, 1998).

### ACKNOWLEDGEMENTS

Our thanks to Janet Squair for statistical advice. Thanks also to the Medical Officers of Health, Tharaka-Nithi, Meru and Ishiolo Districts and Medical Superintendents of Maua, Tigania, Nkubu and Kyeni Hospitals for permission to search patient records.

### REFERENCES

Bayo S, Parkin DM, Koumare AK, Dialo AK, Ba T, Soumare S and Sangare S (1990) Cancer in Mali, 1987–1988. *Int J Cancer* **45**: 679–684

Blaser MJ (1993) Malaria and the natural history of *Helicobacter pylori* infection [letter]. *Lancet* **342**: 551

Cook PJ and Burkitt DP (1971) Cancer in Africa. *Brit Medical Bull* **27**: 14–20

Chokunonga E, Levy LM, Bassett MT, Borok MZ, Mauchaza BG, Chirenje MZ and Parkin DM (1999) Aids and cancer in Africa: the evolving epidemic in Zimbabwe. *AIDS* **13**: 2583–2588

Chokunonga E, Levy LM, Bassett MT, Mauchaza BG, Thomas DB and Parkin DM (2000) Cancer incidence in the African population of Harare, Zimbabwe: second results from the cancer registry. *Int J Cancer* **85**: 54–59

Dent RI (1989) Changes in epidemiology of cancer of the stomach in Zimbabwe [letter; comment]. *Cent Afr J Med* **35**: 493–494

Doll R, Payne P and Waterhouse J, eds. (1966) *Cancer incidence in five continents: a technical report*. Berlin, Springer-Verlag (for UICC)

Doll R, Muir C and Waterhouse J, eds. (1970) *Cancer incidence in five continents* Volume II. Berlin, Springer-Verlag (for UICC)

Echimane AK, Ahnoux AA, Adoubi I, Hien S, M'Bra K, D'Horpock A, Diomande M, Anongba D, Mensah-Adoh I and Parkin DM (2000) Cancer incidence in Abidjan, Ivory Coast. *Cancer* **89**: 653–663

Holcombe C (1992) *Helicobacter pylori*: the African enigma. *Gut* **33**: 429–431

Kitinya JN, Lauren PA, Jones ME and Paljarvi L (1988) Epidemiology of intestinal and diffuse types of gastric carcinoma in the Mount Kilimanjaro area, Tanzania. *Afr J Med Med Sci* **17**: 89–95

Koulibaly M, Kabba ISCA, Diallo SB, Keita N et al. (1997) Cancer incidence in Conakry, Guinea. First results from the cancer registry 1992–1995. *Int J Cancer* **70**: 39–45

Lauren P and Kitinya J (1986) Kilimanjaro Cancer Registry. In: *Cancer occurrence in developing countries*, edited by Parkin, DM. IARC Scientific Publication No. 75, Lyon, p. 108–116

Madebo T, Lindtjorn B and Henriksen TH (1994) High incidence of oesophagus and stomach cancers in the Bale highlands of south Ethiopia. *Trans R Soc Trop Med Hyg* **88**: 415

Newton R, Ngilimana PJ, Grulich A, Beral V, Sindikubwabo B, Nganyira A and Parkin DM (1996) Cancer in Rwanda. *Int J Cancer* **66**: 75–81

Ngendahayo P and Parkin DM (1986) [Cancer in Rwanda. Study of relative frequency]. *Bull Cancer* **73**: 155–164

Parkin DM, Whelan SL, Ferlay J, Raymond L and Young J (1997) *Cancer incidence in five continents Volume VII* IARC Scientific Publication Number 143, Lyon

Parkin DM, Wabinga H and Nambooze S (2001) Completeness in an African cancer registry. *Cancer Causes and Control* **12**: 147–152

Plummer M (1997) Age standardisation. In: *Cancer incidence in five continents volume VII*, edited by Parkin DM, Whelan SL, Ferlay J, Raymond L and Young J. IARC Scientific Publications Number 143, Lyon, p. 66–68

Segal I, Ally R, Sitas F and Walker ARP (1998) *Helicobacter pylori*: the African enigma [letter]. *Gut* **43**: 300–302

Sitas F (1992) *Helicobacter pylori* and mycotoxins in Kenya. In: Sitas F (PhD Thesis). The seroepidemiology of *Helicobacter pylori* and its relationship to chronic atrophic gastritis. Green College, Oxford, p. 227–259

Smith PG (1992) Comparison between registries: age-standardized rates. In: *Cancer incidence in five continents*, 6 edn, edited by Parkin DM, Muir CS, Whelan SL, Gao Y.-T, Ferlay J and Powell J. IARC Scientific Publications, Lyon, p. 865–870

Wabinga HR, Parkin DM, Wabwire-Mangen F and Nambooze S (2000) Trends in cancer incidence in Kyadondo County, Uganda, 1960–1997. *Br J Cancer* **82**: 1585–1592

*© 2001 Cancer Research Campaign*

## References

[PDF_00334] Bayo S., Parkin D. M., Koumaré A. K., Diallo A. N., Ba T., Soumaré S., Sangaré S. (1990). Cancer in Mali, 1987-1988.. Int J Cancer.

[PDF_00336] Blaser M. J. (1993). Malaria and the natural history of Helicobacter pylori infection.. Lancet.

[PDF_00339] Chokunonga E., Levy L. M., Bassett M. T., Borok M. Z., Mauchaza B. G., Chirenje M. Z., Parkin D. M. (1999). Aids and cancer in Africa: the evolving epidemic in Zimbabwe.. AIDS.

[PDF_00342] Chokunonga E., Levy L. M., Bassett M. T., Mauchaza B. G., Thomas D. B., Parkin D. M. (2000). Cancer incidence in the African population of Harare, Zimbabwe: second results from the cancer registry 1993-1995.. Int J Cancer.

[PDF_00338] Cook P. J., Burkitt D. P. (1971). Cancer in Africa.. Br Med Bull.

[PDF_00345] Dent R. I. (1989). Changes in epidemiology of cancer of the stomach in Zimbabwe.. Cent Afr J Med.

[PDF_00351] Echimane A. K., Ahnoux A. A., Adoubi I., Hien S., M'Bra K., D'Horpock A., Diomande M., Anongba D., Mensah-Adoh I., Parkin D. M. (2000). Cancer incidence in Abidjan, Ivory Coast: first results from the cancer registry, 1995-1997.. Cancer.

[PDF_00354] Holcombe C. (1992). Helicobacter pylori: the African enigma.. Gut.

[PDF_00355] Kitinya J. N., Lauren P. A., Jones M. E., Paljarvi L. (1988). Epidemiology of intestinal and diffuse types of gastric carcinoma in the Mount Kilimanjaro area, Tanzania.. Afr J Med Med Sci.

[PDF_00358] Koulibaly M., Kabba I. S., Cissé A., Diallo S. B., Diallo M. B., Keita N., Camara N. D., Diallo M. S., Sylla B. S., Parkin D. M. (1997). Cancer incidence in Conakry, Guinea: first results from the Cancer Registry 1992-1995.. Int J Cancer.

[PDF_00364] Madebo T., Lindtjørn B., Henriksen T. H. (1994). High incidence of oesophagus and stomach cancers in the Bale highlands of south Ethiopia.. Trans R Soc Trop Med Hyg.

[PDF_00367] Newton R., Ngilimana P. J., Grulich A., Beral V., Sindikubwabo B., Nganyira A., Parkin D. M. (1996). Cancer in Rwanda.. Int J Cancer.

[PDF_00369] Ngendahayo P., Parkin D. M. (1986). Le cancer au Rwanda. Etude de fréquence relative.. Bull Cancer.

[PDF_00371] Parkin D. M., Wabinga H., Nambooze S. (2001). Completeness in an African cancer registry.. Cancer Causes Control.

[PDF_00378] Segal I., Ally R., Sitas F., Walker A. R. (1998). Co-screening for primary biliary cirrhosis and coeliac disease. Helicobacter pylori: the African enigma.. Gut.

[PDF_00387] Wabinga H. R., Parkin D. M., Wabwire-Mangen F., Nambooze S. (2000). Trends in cancer incidence in Kyadondo County, Uganda, 1960-1997.. Br J Cancer.

